# D614G mutation in the SARS-CoV-2 spike protein enhances viral fitness by desensitizing it to temperature-dependent denaturation

**DOI:** 10.1016/j.jbc.2021.101238

**Published:** 2021-09-24

**Authors:** Tzu-Jing Yang, Pei-Yu Yu, Yuan-Chih Chang, Shang-Te Danny Hsu

**Affiliations:** 1 Institute of Biological Chemistry, Academia Sinica, Taipei, Taiwan; 2 Institute of Biochemical Sciences, National Taiwan University, Taipei, Taiwan; 3 Academia Sinica Cryo-EM Center, Academia Sinica, Taipei, Taiwan

**Keywords:** SARS-CoV-2, D614G mutation, spike protein, viral protein, protein stability, thermodynamics, cryo-electron microscopy, cold-induced unfolding, vaccine development, 3DVA, three-dimensional variability analysis, ACE2, angiotensin converting enzyme 2, CTF, contrast transfer function, DSC, differential scanning calorimetry, DSF, differential scanning fluorimetry, NSEM, negative staining electron microscopy analysis, RBD, receptor-binding domain, RBM, receptor-binding motif, SARS-CoV-2, severe acute respiratory syndrome coronavirus 2, WT, wild type

## Abstract

The D614G mutation in the spike protein of SARS-CoV-2 alters the fitness of the virus, leading to the dominant form observed in the COVID-19 pandemic. However, the molecular basis of the mechanism by which this mutation enhances fitness is not clear. Here we demonstrated by cryo-electron microscopy that the D614G mutation resulted in increased propensity of multiple receptor-binding domains (RBDs) in an upward conformation poised for host receptor binding. Multiple substates within the one RBD-up or two RBD-up conformational space were determined. According to negative staining electron microscopy, differential scanning calorimetry, and differential scanning fluorimetry, the most significant impact of the mutation lies in its ability to eliminate the unusual cold-induced unfolding characteristics and to significantly increase the thermal stability under physiological pH. The D614G spike variant also exhibited exceptional long-term stability when stored at 37 °C for up to 2 months. Our findings shed light on how the D614G mutation enhances the infectivity of SARS-CoV-2 through a stabilizing mutation and suggest an approach for better design of spike protein-based conjugates for vaccine development.

The COVID-19 (coronavirus disease 2019) pandemic is caused by the infection of SARS-CoV-2 (severe acute respiratory syndrome coronavirus 2) (1). Early bioinformatic analysis of the reported genome sequences of SARS-CoV-2 revealed the emergence of a prominent pairwise linkage disequilibrium between three nucleotide changes since mid-February 2020, namely nt3037 (C > T), nt14408 (C > T), and nt23403 (A > G). The last mutation corresponds to a missense D614G mutation in the spike (S) protein (2). The G clade, which harbors the aforementioned three nucleotide changes and the nt241 (C > T) mutation, relative to the original Wuhan form (hereafter wild type, WT), became the dominant form of the COVID-19 pandemic since the summer of 2020 (https://www.gisaid.org/). The enhanced infectivity of the D614G variant is observed in cell cultures as well as animal models (3).

The SARS-CoV-2 S protein (hereafter S protein) is responsible for host recognition and viral entry. The S protein binds to the receptor, angiotensin converting enzyme 2 (ACE2), through an upward open RBD conformation (RBD-up); a downward closed RBD conformation (RBD-down) sequesters its receptor-binding motif (RBM) from receptor binding, rendering such a conformation inactive (4, 5). Several studies on COVID-19 convalescent sera have identified multiple antibodies that competitively bind to the RBM, and in doing so prevent host receptor ACE2 binding, thereby achieving neutralizing activities (6, 7, 8, 9, 10). Despite the large size, the S protein is marginally stable over a narrow range of temperatures. Incubation of the recombinant SARS-CoV-2 S protein at 4 °C leads to significant unfolding within 24 h. The morphological change of cold denaturation resembles that after a brief heat shock at 50 to 60 °C (11, 12). As a vaccine candidate for mitigating COVID-19, the sensitivity of the recombinant SARS-CoV-2 S protein to cold- and heat-induced unfolding is a major concern. Here, we demonstrated the structural dynamics of the D614G variant spike protein (hereafter S-D614G) by using cryo-EM with the aid of three-dimensional variability analysis (3DVA) (13). Compared with the wild-type S (hereafter S-D614), the increased propensity of S-D614G in the RBD-up conformation implicated the facilitation of the binding to the receptor ACE2. We further conducted the negative staining electronic microscopy (NSEM) analysis coupled with differential scanning calorimetry (DSC) and differential scanning fluorimetry (DSF) to reveal that the D614G mutation eliminates the cold sensitivity of the original D614 and confers the resistance to the high temperature. Our study shows that the S protein with the D614G mutation is more thermally stable than S-D614, suggesting that S-D614G will be a new candidate for vaccine development.

## Results

### S-D614G exhibits abundant conformational heterogeneity with increased propensity of RBD-up conformation

**Figure 1:**
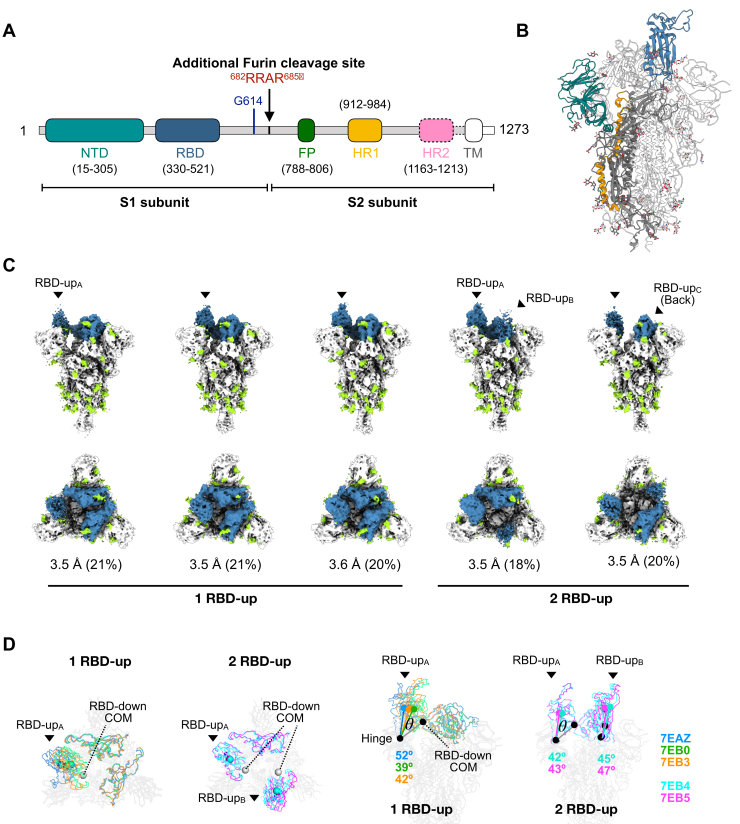
**Cryo-EM analysis of S-D614G.***A*, schematic domain architecture of S-D614G. The regions not resolved in the cryo-EM map are highlighted with *dash line*. *B*, *cartoon* representative of the atomic model of the trimeric S-D614G with the domains colored in accordance with (*A*). *C*, structural heterogeneity of S-D614G. Orthogonal views—*side views* and *top views* are shown on the *top* and *bottom panels*, respectively—of the five distinct clusters of S-D614G derived from 3DVA. The RBDs are colored in *blue*, and the N-glycans are colored in *yellow green*. The nominal resolution of the cryo-EM map and the relative population in percentages are shown below each cluster. *D*, comparison of conformational changes of the RBDs. The structures of one RBD-up and those of two RBD-up were superimposed separately. The RBDs are colored in *blue*, *green*, *orange*, *cyan*, and *magenta* for PDB entries 7EAZ, 7EB0, 7EB3, 7EB4, and 7EB5, respectively. The centers of mass (COMs) of individual RBDs are shown in *sphere* with the matching colors. To calculate the upward rotation angle (θ) of the individual RBD-up conformations, the COM of an RBD-down conformation is generated by aligning the RBD-up protomer structure with respect to the S2 domain of the RBD-up protomer. The hinge is defined as the Cα atom of residue 330 of the RBD-down protomer. The rotation angles (θ) of individual RBD-up conformations are shown in the side views with matching colors. FP, fusion peptide; HR1/HR2, heptad repeat 1/2; NTD, N-terminal domain; RBD, receptor-binding domain; TM, transmembrane domain.

Using cryo-EM single particle reconstruction aided by 3DVA analysis (13, 14), we identified five distinct but equally populated clusters of conformations of S-D614G with varying degrees of RBD-up populations (Fig. 1, Fig. S1 and Table S1). Collectively, 62% of the total population of S-D614G had one RBD in an up conformation (one RBD-up), including three distinct substates that separated the RBD conformations, and 38% of the population had two RBDs in an up conformation (two RBD-up) with two different substates. The three distinct RBD-up substrates in the one RBD-up class showed a 39 to 52° upward rotation with respect to the hinge defined as the Cα atom of the beginning of RBD, *i.e.*, residue 330 (Fig. 1*D*). Similarly, the two distinct RBD-up substates in the two RBD-up class showed a 42 to 47° upward rotation. Contrary to S-D614, we did not observe any all RBD-down conformation in S-D614G, in line with the previous study of a similar construct without the transmembrane domain (15), but differs from the full-length S-D614G that exhibits significant amount of closed, all RBD-down conformation (16, 17). Two recent studies reported several cryo-EM structures of S-D614G with or without the furin cleavage site mutations (fm) and/or the tandem proline stabilization mutation (2P, see Experimental procedures), which showed either one RBD-up or all RBD-down conformations (18, 19). Our study is one of the only two, in addition to Benton *et al.* (15), that reported the two RBD-up conformation. Although Yurkovetskiy *et al.* (20) reported an all RBD-up conformation of S-D614G, the EM density of the RBDs is poorly defined. A common feature of reported S-D614G structures, however, is the increased propensity to populate the RBD-up conformations, and the two RBD-up conformations have not been reported for S-D614 (Table S2). Since the upward conformation of the RBD is the prerequisite of host receptor ACE2 binding, the increased population of the RBD-up conformations with more conformational plasticity may explain why S-D614G is found to exhibit enhanced binding to the receptor ACE2 (21).

Yurkovetskiy *et al.* (20) proposed that the D614G mutation may disrupt the interprotomer hydrogen bond between D614 of one protomer and T859 of the other protomer, thereby leading to increase dynamics of the S protein and the shift of equilibrium between the RBD-up and RBD-down. However, our cryo-EM structure of S-D614G showed no appreciable structural difference in the proximity of the mutation site with respect to some of the extremities of the reported S-D614 structures, including the first reported structures of S-D614 (4, 5), the acid-stabilized form of S-D614 (22), the disulfide- and proline-stabilized HexPro-S variant (23), and the furin-cleaved S-D614 that adopts a more open conformation (Figs. S2-S4) (24). Meanwhile, Benton *et al.* (15) proposed that the loss of a key salt bridge formed by D614 of one protomer and K584 or the other protomer due to the D614G mutation leads to local disorder, thereby increasing the dynamics of S-D614G and more populated RBD-up conformations, a finding that could be confirmed by our cryo-EM structures (Fig. S5). Nevertheless, the molecular basis of how the D614G mutation could allosterically change the conformation and dynamics of the RBDs remains to be established.

### The D614G mutation enhances the thermal stability of S protein at wide range of temperature

**Figure 2:**
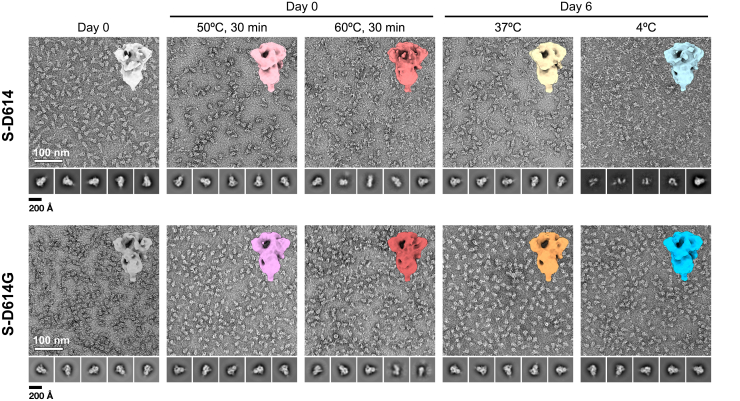
**S-D614G is highly stable over a broad range of temperatures.** Representative NSEM micrographs of S-D614 (*top panels*) and S-D614G (*bottom panels*) after different temperature treatments. Selected 2D classes of picked particle images are shown below each panel. All micrographs and 2D classes are shown in the same scales. The scale bars of the micrograph and the 2D classes are indicated on the *lower left* corners of the Day 0 datasets. Significant unfolding of S-D614 was observed after heat shocks and after 6 days of incubation at 4 °C. In contrast, S-D614G exhibited visible unfolding only by heat shock at 60 °C for 30 min 3D maps of the individual samples derived from the native-like particle images are shown on the *upper right* corner of each panel.

Having established that the structure of S-D614G is not significantly perturbed by the D614G mutation under native conditions, we sought to investigate the impact of the D614G mutation on the thermal stability over a range of experimental conditions. Knowing that the S protein is susceptible to cold denaturation, all experiments were carried out using freshly prepared S-D614 and S-D614G, which were secreted into the culture media at 37 °C, and purified at room temperature (Fig. S6). We carried out negative stain electron microscopy (NSEM) analyses on S-D614 and S-D614G on day 0 and on day 6 after continuous incubation at 37 °C and 4 °C. In line with the previous finding (12), S-D614 remained stable at 37 °C for 6 days without discernible morphological change, while significant unfolding was observed after incubation at 4 °C for 6 days (Fig. 2).

**Figure 3:**
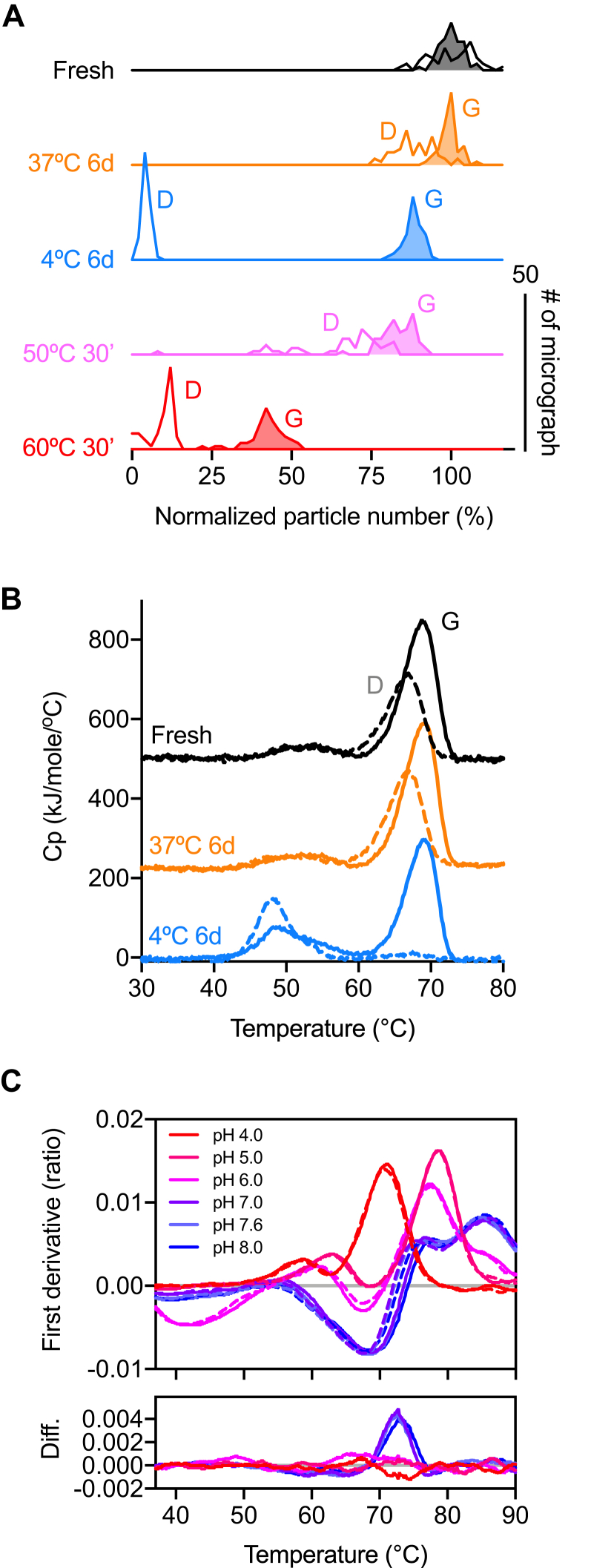
**Quantitative analyses of the thermal stabilities of S-D614 and S-D614G.***A*, histograms of the relative amounts of native-like particle images with respect to the fresh samples. The Y-axis corresponds to the number of micrographs (n = 50–60). The *open* and *filled curves* correspond to S-D614 and S-D614G, respectively. *B*, DSC profiles S-D614 (*dashed lines*) and S-D614G (*solid lines*) at Day 0 (fresh; *black*), 37 °C for 6 days (*orange*) and 4 °C for 6 days (*light blue*). *C*, DSF profiles of S-D614 (*dashed lines*) and S-D614G (*solid lines*) as a function of pH values. The difference between S-D614G and S-D614 (Diff.) is derived by subtracting the values of S-D614 by those of S-D614G.

**Figure 4:**
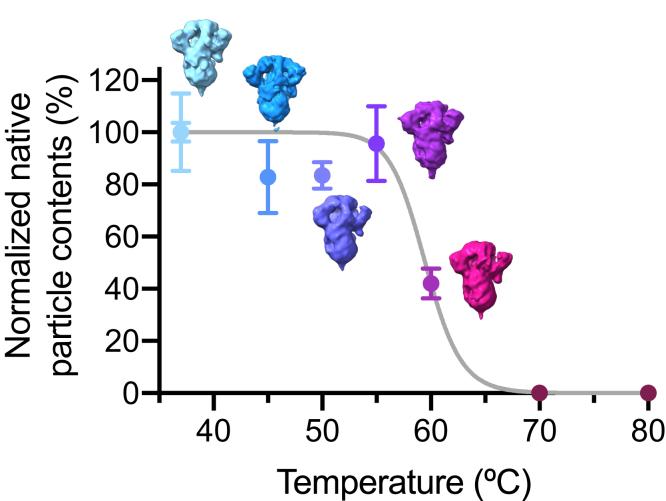
**Relative native-like particle number of S-D614G as a function of temperature.** All samples were incubated at the specified temperatures as indicated along the X-axis for 30 min prior to NESM grid preparation. The numbers of native-like particles in individual micrographs were normalized with respect to the average value of freshly prepared sample (37 °C, day 0). The error bars correspond to the standard deviations of the numbers within the micrographs collected under the same conditions. For each temperature, between 50 and 60 NSEM micrographs were collected. 3D maps of the individual samples derived from the native-like particle images are shown next to the data points.

Quantitative analysis showed that the 6-day incubation at 37 °C and 4 °C resulted in 12 ± 6% and 96 ± 1% loss of native particles, respectively (Figs. 2 and 3*A*, Table S3). Unexpectedly, S-D614G did not show significant unfolding after the same cold treatment: the 6-day incubation at 37 °C and 4 °C resulted in 1 ± 3% and 12 ± 3% loss of native particles, respectively (Figs. 2 and 3*A*). Remarkably, after 2 months of incubation at 37 °C, S-D614G remained mostly intact according to NSEM analysis (Fig. S7). When subjected to heat-induced denaturation of both proteins at 50 °C and 60 °C, S-D614 exhibited significant unfolding at 30 min of heat shock at 50 °C and 60 °C, resulting in 34 ± 15% and 91 ± 4% loss of native particles, respectively. In contrast, the same heat shock treatment resulted in 17 ± 5% (50 °C) and 58 ± 6% (60 °C) loss of native particles for S-D614G. Furthermore, S-D614 exhibited more variations in the number of native particles per micrograph, particular in the case of heat treatment at 50 °C for 30 min (Fig. 3*A*). Note, however, that despite the significant reduction in the number of native-like particles after cold and/or heat treatments, the resulting 3D EM maps of S-D614 and S-D614G were very similar at the resolution of approximately 10 Å (insets in Fig. 2), suggesting that the unfolding processes of the S protein variants follow an apparent two-state model resulting in different levels of native populations after the treatments. Indeed, further analysis with more incubation temperatures for S-D614G yielded a two-state-like melting curve with an apparent T_m_ of 59 °C (Fig. 4).

We further analyzed the thermal unfolding of S-D614 and S-D614G at pH 7.6 by DSC. Both S-D614 and S-D614G exhibited two transition peaks and shared a similar melting temperature (T_m_) for the first transition at *ca.* 52 °C. However, S-D614G exhibited a higher T_m_ for the second transition (T_m_ = 68.8 °C) compared with that of S-D614 (T_m_ = 66.9 °C). Moreover, the total enthalpy of unfolding ΔH of S-D614G was approximately 40% higher than that for S-D614 (Fig. 3*B* and Table 1). The bimodal distribution of the DSC profile of S-D614G has been reported previously (12, 22), and that the second transition was missing in some cases; this may be attributed to prolonged sample storage at lower temperatures (22). Indeed, the second transition peak was lost for S-D614 after incubation at 4 °C for 6 days, while that of S-D614G remained largely unaffected; in line with NSEM results, long-term incubation at 37 °C did not significantly affect both variants (Fig. 3*B*). While keeping purified proteins at 4 °C for long-term storage is generally considered to be more favorable than storage at 37 °C, this was not applicable to S-D614. In contrast, S-D614G was not sensitive to cold denaturation and is more robust during transient heat shock (Table 1). The stabilization effect of the D614G mutation was most pronounced under neutral to slightly alkaline conditions as demonstrated by label-free DSF analyses of S-D614 and S-D614G over a range of pH values (Fig. 3*C*). Both variants exhibited multiple inflection temperatures (T_i_) between pH 4 and 8 as has been reported previously (12, 22). The complex melting curves indicated the presence of multiple transition events sensed by the large number of tryptophans and tyrosines within S-D614 and S-D614G, but it is not trivial to assign a specific transition point to a given tryptophan or tyrosine residue. Nonetheless, pairwise comparison of the unfolding curves of S-D614 and S-D614G showed that their profiles were indistinguishable at pH 6 and below. For pH 7.0 and above, only the transition between 70 and 75 °C showed a clear difference between the two variants, suggesting that the stabilizing effect of D614G is likely contributing to the integrity of the S protein before being endocytosed into the host cell.

**Table 1:** Summary of DSC analysis of S-D614 and S-D614G after different treatments

Condition	Sample	Enthalpy of unfolding (kcal mol^−1^)	T_m1_ (°C)	T_m2_ (°C)
Fresh	S-D614	1580		66.9
	S-D614G	2250		68.8
37 °C, 6 days	S-D614	997		67.0
	S-D614G	1560		68/9
4 °C, 6 days	S-D614	853	48.2	66.8
	S-D614G	2120	49.0	68.9

## Discussion

In this work, we determined the cryo-EM structure of S-D614G and revealed the high degree of conformational heterogeneity mostly confined within the RBDs (Fig. 1*C*). Contrary to the previous report by Yurkovetskiy *et al.* (20), we did not observe appreciable conformational rearrangement around the interprotomer interaction site between D614 and T859, but we did observe significant disorder of the K854 loop as reported by Benton *et al.* (Fig. S4) (15). The most significant finding is the markedly increased resistance of S-D614G to withstand cold- and heat-induced unfolding (Fig. 2). Although the loss of the salt bridge between D614 and K584 is enthalpically unfavorable, it is compensated by other enthalpic gains evidenced by the significantly higher total enthalpy of unfolding ΔH of S-D614G compared with that of S-D614 (Fig. 2). We argue that the increased stability of S-D614G could be further attributed to the increased configurational entropy manifested in the higher conformational heterogeneity of the RBDs (Fig. 1*C*), which contributes to the free energy gain of the system.

The significance of our findings is threefold. First, the increased folding stability may help explain the gain in fitness of the G clade SARS-CoV-2, which relies on the surface S protein to recognize host receptor molecules, namely ACE2. The elimination of the cold sensitivity of the S protein will undoubtedly increase the robustness of the infection machinery over a range of environment conditions. Second, our DSC analysis of freshly prepared S protein variants hinted at the possibility that some of the previously reported cryo-EM and biophysical studies of the SARS-CoV-2 S protein may have been affected by its sensitivity to cold treatments leading to the unfolding and the loss of structural elements that give rise to the thermal transition peaks at around 60 to 70 °C (Fig. 2). More importantly, the partial unfolding of the S protein is expected to significantly alter the accessibility of the RBD to which ACE2 and neutralizing antibodies bind and hence impact interpretations of binding assays. Third, the ability of S-D614G to withstand long-term storage at 4 °C without unfolding the prefusion state provides a solution to better vaccine designs and formulation without the need to introduce a large number of mutations and disulfide bonds (11, 23, 25).

## Experimental procedures

### Expression and purification of SARS-CoV-2 S and its variant, S-D614G

The codon-optimized nucleotide sequence of full-length SARS-CoV-2 S protein (UniRule annotation HAMAP-Rule:MF_04099) was a kind gift of Dr Che Alex Ma (Genomics Research Center, Academia Sinica). The DNA sequence corresponding the residues 1 to 1208 of the S protein was subcloned into the mammalian expression vector pcDNA3.4-TOPO (Invitrogen). Additionally, a tandem proline mutation (2P, ^986^KV^987^ → ^986^PP^987^) and changes at the furin cleavage site (fm, ^682^RRAR^685^ → ^682^GSAG^685^) were introduced for stabilization (4), which corresponds to S-D614. The D614G mutation was subsequently introduced to generate S-D614G. Al foldon trimerization domain based on phage T4 fibritin followed by a c-myc epitope and a hexa-repeat histidine tag were introduced to the C-termini of both S-D614 and S-D614G as described previously (26). The exact sequences of S-D614G and S-D614 are included in the Protein Data Bank (PDB) entries of the reported structures. This study reported five structures of S-D614G under the PDB entries 7EAZ, 7EB0, 7EB3, 7EB4, and 7EB5. The protein sequence of S-D614 is described in the PDB entries 7EJ4 and 7EJ5.

The plasmids of S-D614 and S-D614G were transiently transfected into HEK293 Freestyle cells with polyethylenimine (PEI, linear, 25 kDa, Polysciences). The transfected cells were incubated at 37 °C with 8% CO_2_ for 6 days. After pelleting cells by centrifugation at 4000 rpm for 30 min, the medium was harvested and incubated with HisPur Cobalt Resin (Thermo Fisher Scientific) in binding buffer (50 mM Tris-HCl (pH 7.6), 300 mM NaCl, 5 mM imidazole and 0.02% NaN_3_) at 4 °C overnight. The resin was washed with wash buffer (50 mM Tris-HCl (pH 7.6), 300 mM NaCl, 10 mM imidazole), and the target protein was eluted by elution buffer (50 mM Tris-HCl (pH 7.6), 150 mM NaCl, 150 mM imidazole). The protein was concentrated and loaded into a size-exclusion chromatography (SEC) column (Superose 6 increase 10/300 GL; GE Healthcare) with a running buffer (50 mM Tris-HCl (pH 7.6), 150 mM NaCl, 0.02% NaN_3_) for further purification. The protein concentrations were determined by using the UV absorbance at 280 nm using an UV-Vis spectrometer (Nano-photometer N60, IMPLEN).

### Cryo-EM sample preparation of S-D614G and data collection

Three microliters of purified protein was applied onto 300-mesh Quantifoil R1.2/1.3 holey carbon grids. The grids were glow-charged at 20 mA for 30 s. After 30-s incubation, the grids were blotted for 2.5 s under 4 °C with 100% humidity and vitrified using a Vitrobot Mark IV (Thermo Fisher Scientific). Data acquisition was performed on a 300 keV Titan Krios microscope equipped with a Gatan K3 direct detector (Gatan) in a super-resolution mode using the EPU software (Thermo Fisher Scientific). The cryo-EM data were collected in a movie mode with a defocus range of −0.8 to −2.6 μm at a magnification of 81,000×, which corresponded to a pixel size of 0.55 Å. A total dose of 48 e^−^/Å^2^ was distributed over 50 frames with an exposure time of 1.8 s. The dataset was collected with an energy filter (slit width: 15–20 eV), and the dose rate was adjusted to 8 e^−^/pix/s.

### Image processing and 3D reconstruction

All 2× binned super-resolution movies were processed by Relion-3.0 (27) with dose-weighting and 5 × 5 patch-based alignment using the GPU-based software MOTIONCOR2 (28). All motion-corrected micrographs were further processed by cryoSPARC v2.14 (29). Contrast transfer function (CTF) estimation was performed by patch-based CTF. The micrographs that were considered by the script “CTF_fit_to_Res” as satisfactory (between 2.5 and 4 Å) were used for particle picking. A small subset of micrographs was used for unbiased template-free blob picker within cryoSPARC. The picked particles were extracted with a box size of 192 pixels (2 × 2-binned), followed by iterative 2D classifications with visual inspections to remove junk particles.

To identify conformational heterogeneity of S-D614G trimer, ∼660k particles were initially classified by *ab-initio* reconstruction with a C1 symmetry, followed by heterogeneous refinement to generate five distinct classes. One of the 3D classes showed a single RBD-up conformation (388,400 particles), whereas the remaining classes showed poorly defined cryo-EM maps, which were excluded from the subsequent data process. The particles that corresponded to the single RBD-up conformation were used for further processing by using nonuniform 3D refinement (NU-refinement) with a C1 symmetry. This yielded a 3.1 Å cryo-EM map according to the Fourier shell correlation (FSC) = 0.143. The map and mask from the NU-refinement were used for the 3D variability analysis (3DVA) as part of cryoSPARC, which generated five clusters for further heterogeneous refinement. The particle images from each 3D class were unbinned and re-extracted with a box size of 384 pixels. The full-resolution particle stacks were used for the NU-refinement, local CTF refinement, a second round of NU-refinement to generate five final cryo-EM maps, including three “one RBD-up” conformations (3.5, 3.6, and 3.6 Å) and two “two RBD-up” conformations (3.5 and 3.4 Å). The local resolution analysis for was calculated using ResMap (30).

### Model building and refinement

An initial model was generated by Swiss-Model (31) using the PDB structure 6XM3 as a template. The atomic coordinates were divided into individual domains and manually fit into the cryoEM maps of S-D614G by using UCSF-Chimera (32), UCSF-ChimeraX (33) and Coot (34). After iterative refinements, the structural models were refined by the real-space refinement module within Phenix (35). Twenty two previously reported N-glycosylation sites were examined further to identify additional cryo-EM densities protruding from the corresponding asparagine side chain, which implied the presence of N-glycan moieties. In cases where additional EM densities were clearly visible, atomic models of N-linked glycans were built onto the asparagine side chains by using the module “Glyco” within Coot (34). The final model was assessed by MolProbity (36). Structural visualization and rendering of structural representations were achieved by using a combination of UCSF-Chimera, UCSF-ChimeraX, and Pymol (Schrodinger Inc).

### Negative staining electron microscopy analysis (NSEM)

Four microliters of S-D614 and S-D614G that were treated at different temperatures and durations—fresh (day 0), fresh samples incubated at 50 °C/60 °C for 30 min, and fresh samples incubated at 4 °C/37 °C for 6 days—were used to prepare negative staining EM grids at a concentration of 50 μg/ml. The carbon-coated grids were glow-discharged with 25 mA for 30 s. After staining with 0.2% uranyl formate, the grids were blotted and dried at the air for 1 day. Images were collected by using a FEI Tecnai G2-F20 electron microscope at 200 keV (FEI). A magnification of 50,000× was used, corresponding to a pixel size of 1.732 Å. All datasets were processed by cryoSPARC v2.14, including patch-CTF estimation, particle picking/extraction, 2D classification, and *ab-initio* 3D reconstruction. The number of “intact” spike particles in each micrograph (defined by the number of particles used in 3D reconstruction) was extracted by the function “Manually curate exposures” within cryoSPARC. The numbers were exported to GraphPad Prism 8 (GraphPad) for further statistical analyses. The 3D models of individual experimental conditions were visualized by using UCSF-ChimeraX.

### Differential scanning calorimetry (DSC)

The thermal unfolding of S-D614 and S-D614G was analyzed by DSC (Automatic MicroCal PEAQ-DSC). The protein concentrations were set to 0.2 mg/ml in 50 mM Tris-HCl (pH 7.6), 150 mM NaCl, and 0.02% NaN_3_. The temperature was ramped up from 10 to 90 °C at a rate of 200 °C/h. The resulting data were baseline-corrected by the built-in software of MicroCal PEAQ-DSC and exported to GraphPad Prism 8 to generate the graphs.

### Differential scanning fluorimeter (DSF)

All experiments were performed in Tycho NT.6 (NanoTemper Technologies). Fresh samples, from biological replicates, were ten times diluted in 100 mM sodium acetate (pH 4.0 and pH 5.0), MES (pH 6.0), HEPES (pH 7.0), or Tris (pH 7.6 and pH 8.6) supplement with 150 mM NaCl at the final concentration of 0.15 mg/ml. The temperature range was set to 35 to 95 °C, and the scanning rate was 30 °C/min. All data were processed in the internal software from NanoTemper, and the statistics of experimental parameters analyzed by GraphPad Prism 8.

## Data availability

Experimental materials and methods, including molecular cloning, protein expression and purification, NSEM and cryo-EM, DSF and DSC, are described in Supporting information. The atomic coordinates of the S-D614G are deposited in the PDB under the accession codes: 7EAZ, 7EB0, 7EB3, 7EB4, and 7EB5. The corresponding cryo-EM maps are deposited in the Electron Microscopy Data Bank (EMDB) under accession codes: EMD-31047, 31048, 31050, 31051, and 31052.

## Supporting information

This article contains supporting information.

## Conflict of interest

The authors declare that they have no conflicts of interest with the contents of this article.
