# Supplementary material for: D614G mutation in the SARS-CoV-2 spike protein enhances viral fitness by desensitizing it to temperature-dependent denaturation
Source: J Biol Chem. 2021 Sep 24;297(4):101238. doi: 10.1016/j.jbc.2021.101238 (PMC8460419; doi:10.1016/j.jbc.2021.101238)
Supplement: Supplemental Figures S1–S7 and Tables S1–S3 [file mmc1.pdf]

Supporting information for

D614G mutation in the SARS-CoV-2 spike protein enhances viral fitness by desensitizing it to temperature-dependent denaturation

Tzu-Jing Yang[1,2], Pei-Yu Yu[1], Yuan-Chih Chang[1,3], and Shang-Te Danny Hsu[1,2,*]

1. Institute of Biological Chemistry, Academia Sinica, Taipei 11529, Taiwan
2. Institute of Biochemical Sciences, National Taiwan University, Taipei 10617, Taiwan
3. Academia Sinica Cryo-EM Center, Academia Sinica, Taipei 11529, Taiwan

Correspondence to: sthsu@gate.sinica.edu.tw

This PDF file includes:

Figs. S1 to S7

Tables S1 to S3

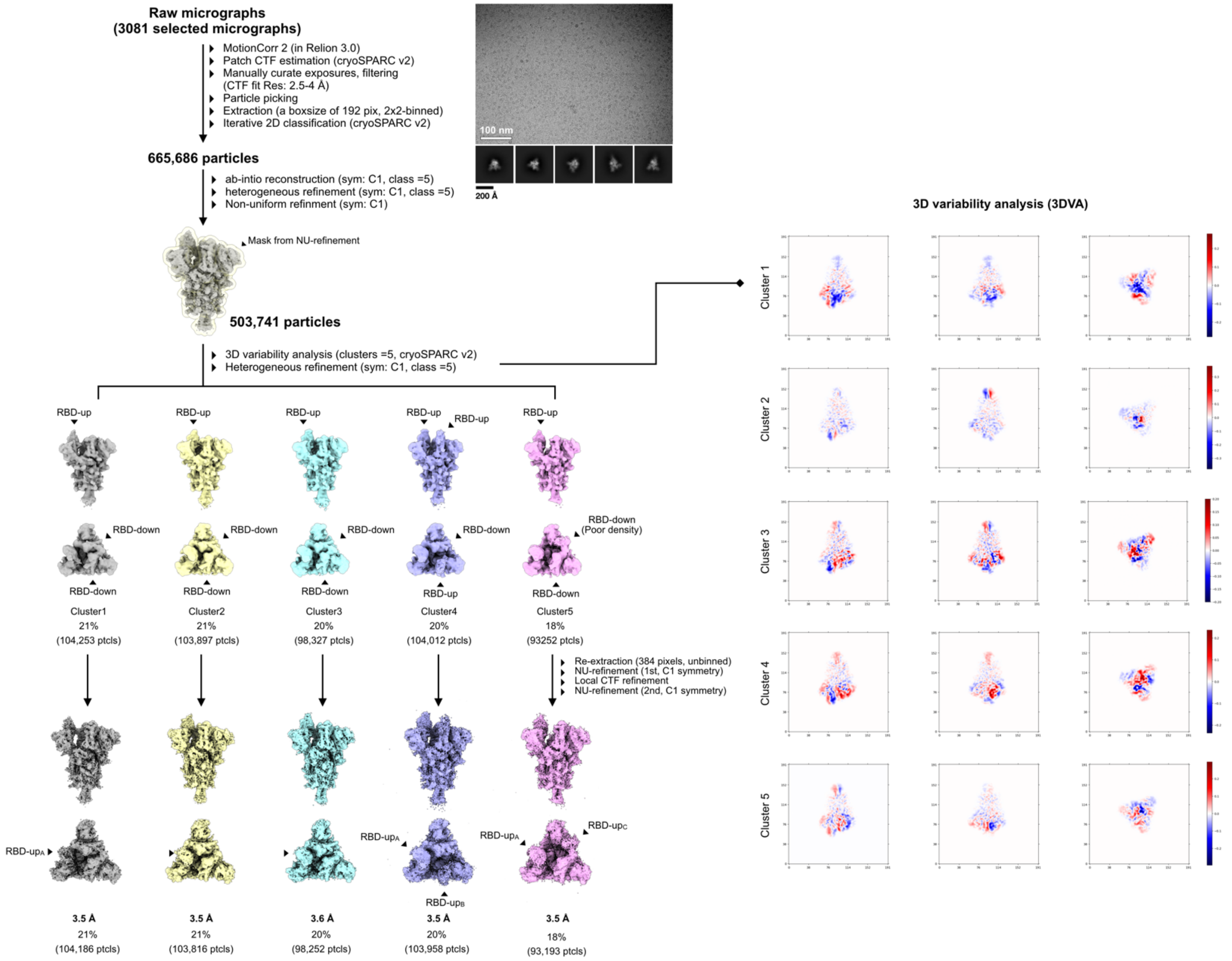

Fig. S1. Workflow for cryo-EM data processing.

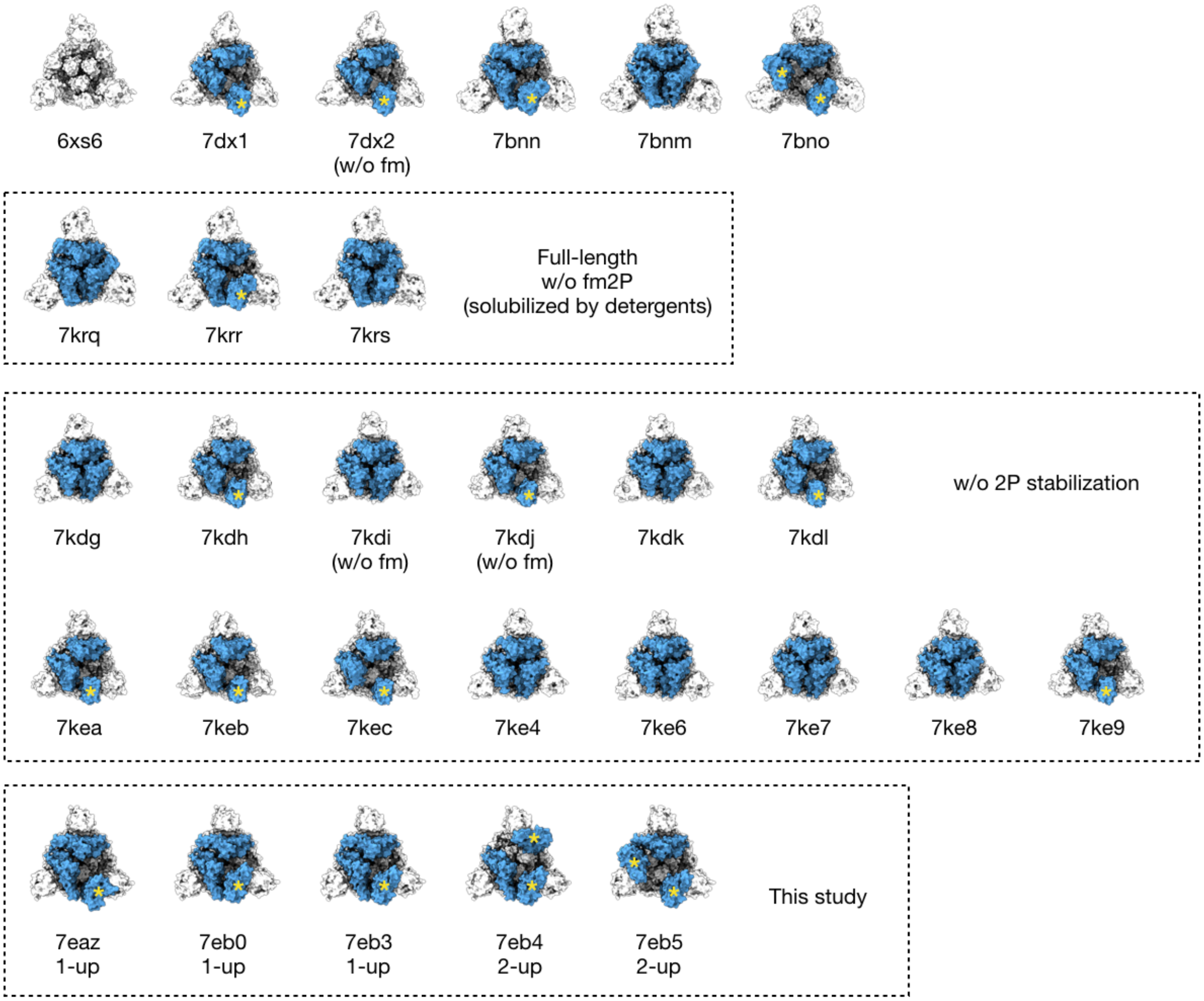

Fig. S2. Comparison of reported cryo-EM structures of S-D614G. Top views of individual cryo-EM structures with the RBD (residues 330-521) colored in blue. The RBDs that are in the up conformations are indicated by yellow asterisks. Details of the constructs designs of individual structures are tables in Table S2. Note that the PDB entry 6xs6 lacks resolved EM density for the RBD, but it was concluded by the authors that all three RBDs are in the up conformation.

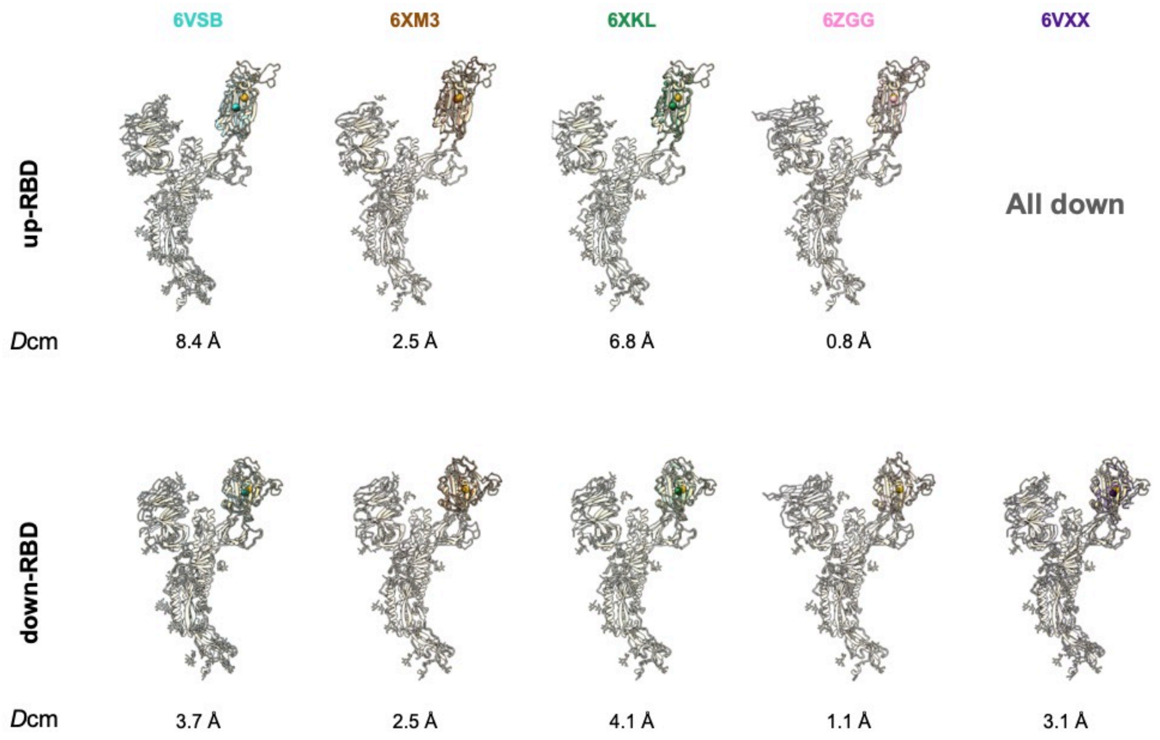

Fig. S3. Comparison of RBD orientations of reported S-D614G and S-D614 structures. Cartoon representations of RBD-up and RBD-down protomers derived from previously reported cryo-EM structures and our current study. The PDB accession codes of individual structures are shown above. The PDB entries of the representative spike variants are 6VSB (S-D614 in an 1 RBD-up), 6VXX (all RBD-down, closed state), 6XM3 (1 RBD-up, at pH 5.5), 6XKL (engineering thermal-stable mutant, HexaPro, 1 RBD-up), and 6ZGG (furin-cleaved, one RBD-up). All protomers were superimposed with respect to the S2 domains of S-D614G of this study. After the alignment with respect to the S2 domain, the displacement between the centers of mass (Dcm; indicated by spheres) of previously reported RBD structure and ours is calculated by UCSF-Chimera, which is indicated below each structure.

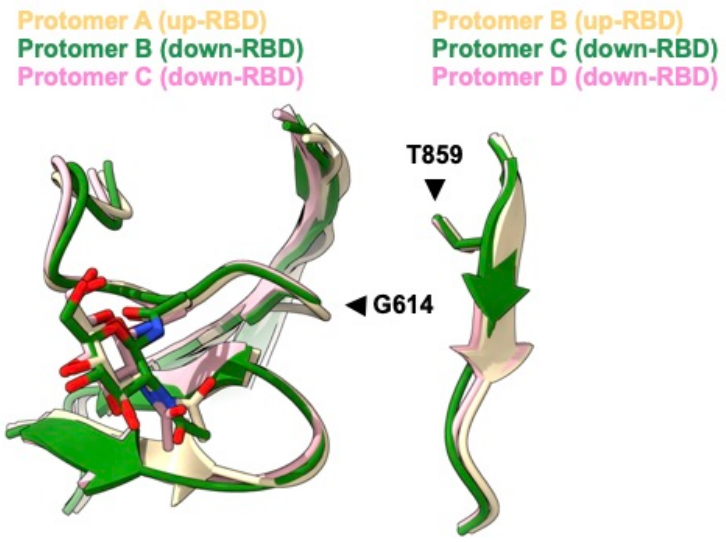

Fig. S4. Superposition of the structural elements surrounding the D614G mutation site from within the three protomers (chains) of S-D614G (chains A, B, and C). The results indicated that local structures of the mutation site are essentially identical, despite the difference of their corresponding RBD orientations. The protomers are colored in wheat (protomer A, RBD-up), green (protomer B, RBD-down), and pink (protomer C, RBD-down), respectively. The first GlcNAc moiety that is linked to the side chain of N616 is shown in sticks.

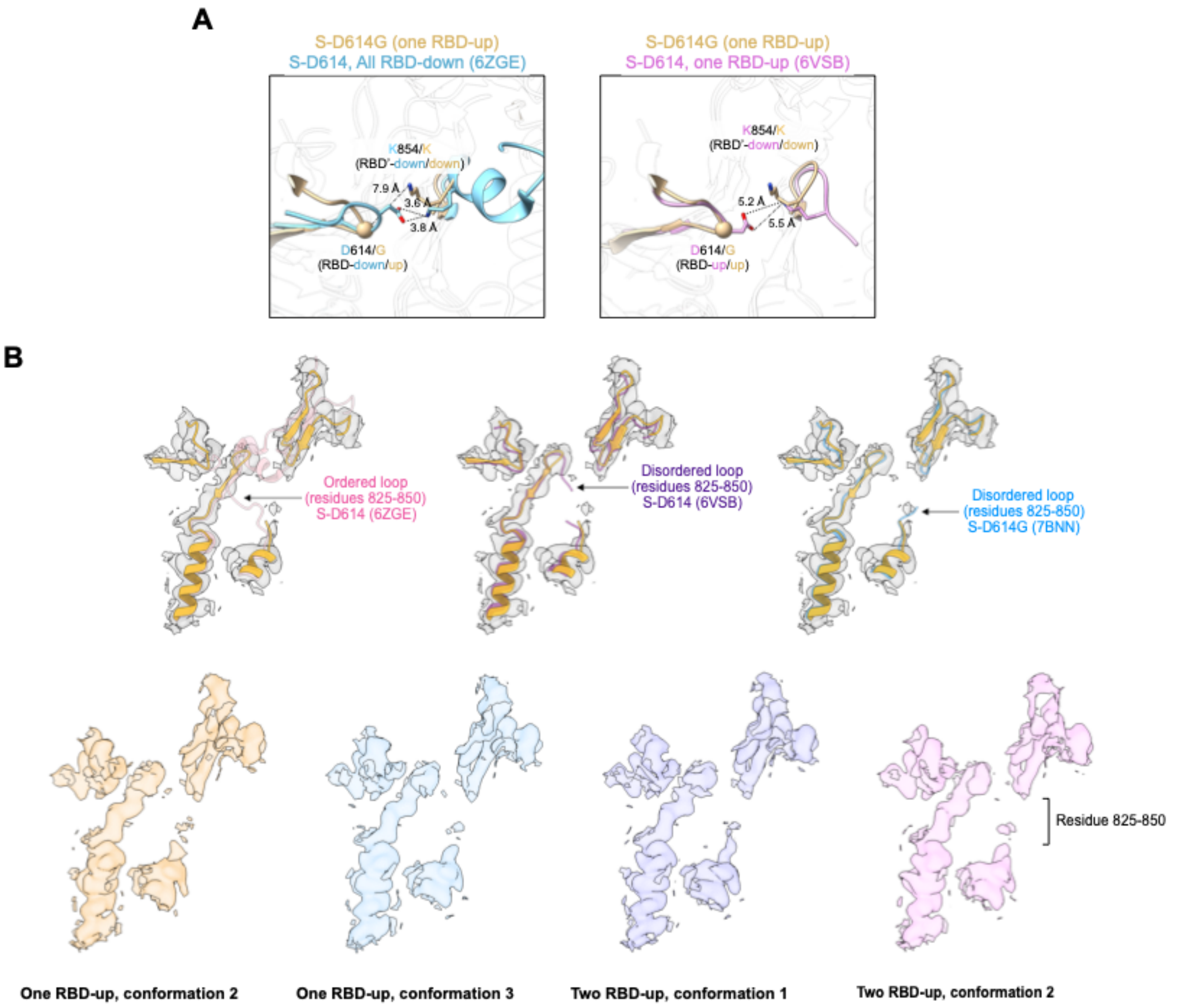

Fig. S5. Conformational disorder around the D614G mutation site. (A) Structural comparison of the inter-protomer interaction between S-D614G and S-D614 (two conformations: all RBD-down and one RBD-up). The dash lines indicate the distance between G614 (or D614) and K854. The structure of S-D614G is colored in tan. The structures of S-D614 with all RBD-down (PDB code: 6ZGE; left panel) and with one RBD-up (PDB code: 6VSB; right panel) are colored in blue and pink, respectively. (B) The loop on which K584 resides is disordered in all the selected cryo-EM structures, which is manifested in the lack of defined cryo-EM maps for residues 825-850 as indicated on the right.

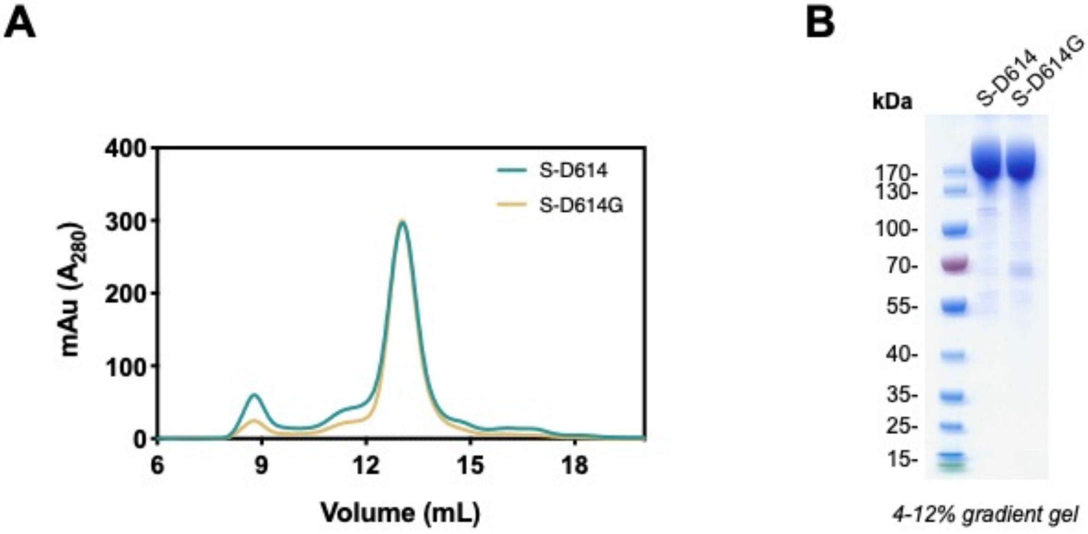

Fig. S6. Purification of recombinant S-D614 and S-D614G. (A) Overlaid SEC chromatograms of S-D614 (grass green) and S-D614G (yellow). (B) Coomassie Brilliant Blue stained SDS-PAGE, which illustrated the purity and integrity of the purified recombinant S-D614 and S-D614G.

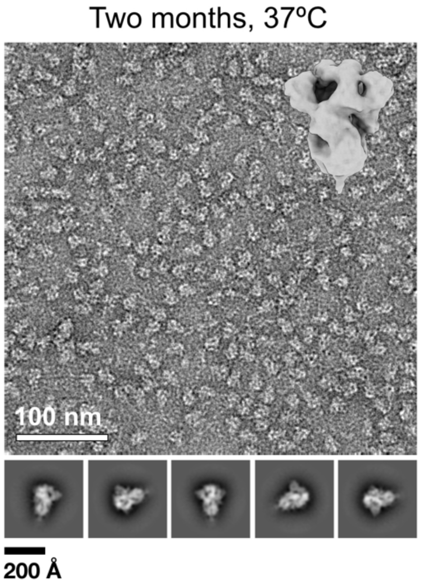

Fig. S7. Representative NSEM image and 2D classes of S-D614G incubated at 37 ºC for 60 days. The 3D model derived from the NSEM data was shown on the upper right corner.

.

Table S1. Parameters of cryo-EM data collection, processing and model validation.

	1 RBD-up conformation 1	1 RBD-up conformation 2	1 RBD-up conformation 3	2 RBD-up conformation 1	2 RBD-up conformation 2
**Data collection and processing**					
Microscope			Titan Krios (Gatan K3 Summit camera)		
Voltage (keV)			300		
Mode			Counting		
Magnification			81,000x		
Total dose ($e^-/Å^2$)			48		
Defocus range (μm)			0.8-2.6		
Pixel size (Å)			1.1 (2x binned)		
Final used particles	104,186	103,816	98,252	103,958	93,193
Symmetry	C1	C1	C1	C1	C1
Map Resolution (Å)	3.5	3.6	3.6	3.5	3.4
**Model composition**					
Non-hydrogen atoms	24,959	24,949	24,847	24,835	24,905
Protein residues	3,051	3,055	3,041	3,047	3,058
Ligands	82	80	79	75	74
MolProbity score	1.81	1.79	1.85	1.74	1.79
Ramachandran (%)					
Favored	94.67	94.08	93.61	95.20	94.42
Allowed	5.29	5.88	6.32	4.77	5.44
Outliners	0.03	0.03	0.07	0.03	0.13
Rotamer outliners (%)	0.00	0.00	0.04	0.00	0.00
Clashscore	8.21	7.12	7.90	7.35	7.52
r.m.s. deviations					
Bond length (Å)	0.003	0.003	0.004	0.003	0.003
Bond angles (º)	0.600	0.586	0.593	0.557	0.573
**Data Deposition**					
PDB code	7EAZ	7EB0	7EB3	7EB4	7EB5
EMDB code	31047	31048	31050	31051	31052

Table S2. Comparison of reported cryo-EM structure of S-D614G

<table>
<tr><th>PDB ID</th><th>RBD conformation</th><th>C-terminal affinity tag</th><th>Furin cleavage site mutation</th><th>2P stabilization mutation</th><th>Others</th><th>Ref</th></tr>
<tr><td>6XS6</td><td>3 RBD-up</td><td>His-tag</td><td>✓</td><td>✓</td><td></td><td>(1)</td></tr>
<tr><td>7DX1</td><td>1 RBD-up</td><td rowspan="2">Flag tag</td><td rowspan="2">✓</td><td rowspan="2">✓</td><td rowspan="2"></td><td rowspan="2">(2)</td></tr>
<tr><td>7DX2</td><td>1 RBD-up</td></tr>
<tr><td>7KRQ</td><td>All-down</td><td rowspan="3">Twin Strep tag</td><td rowspan="3"></td><td rowspan="3"></td><td rowspan="3">Full-length construct solubilized by detergents</td><td rowspan="3">(3)</td></tr>
<tr><td>7KRR</td><td>1 RBD-up</td></tr>
<tr><td>7KRS</td><td>1 RBD-up (intermediate)</td></tr>
<tr><td>7BNM</td><td>All-down</td><td rowspan="3">His-tag</td><td rowspan="3">✓</td><td rowspan="3">✓</td><td rowspan="3"></td><td rowspan="3">(4)</td></tr>
<tr><td>7BNN</td><td>1 RBD-up</td></tr>
<tr><td>7BNO</td><td>2 RBD-up</td></tr>
<tr><td>7KDG</td><td>All-down</td><td rowspan="15">Twin Strep tag and 8xHis tag</td><td>✓</td><td rowspan="15"></td><td rowspan="15"></td><td rowspan="15">(5)</td></tr>
<tr><td>7KDH</td><td>1 RBD-up</td><td>✓</td></tr>
<tr><td>7KDI</td><td>All-down</td><td rowspan="2"></td></tr>
<tr><td>7KDJ</td><td>1 RBD-up</td></tr>
<tr><td>7KDK</td><td>All-down</td><td>✓</td></tr>
<tr><td>7KDL</td><td>1 RBD-up</td><td>✓</td></tr>
<tr><td>7KEA</td><td>1 RBD-up</td><td>✓</td></tr>
<tr><td>7KEB</td><td>1 RBD-up</td><td>✓</td></tr>
<tr><td>7KEC</td><td>1 RBD-up</td><td>✓</td></tr>
<tr><td>7KE4</td><td>All-down</td><td>✓</td></tr>
<tr><td>7KE6</td><td>All-down</td><td>✓</td></tr>
<tr><td>7KE7</td><td>All-down</td><td>✓</td></tr>
<tr><td>7KE8</td><td>All-down</td><td>✓</td></tr>
<tr><td>7KE9</td><td>1 RBD-up</td><td>✓</td></tr>
</table>

Table S3. Parameters of negative stain electron microscopy (NSEM) data collection and processing.

	S-D614					S-D614G					
	Day 0	Day 0<br>50ºC<br>30 min	Day 0<br>60ºC<br>30 min	Day 6<br>37ºC	Day 6<br>4ºC	Day 0	Day 0<br>50ºC<br>30 min	Day 0<br>60ºC<br>30 min	Day 6<br>37ºC	Day 6<br>4ºC	Day 60<br>37ºC
**Data collection**											
Microscope	Tecnai G2-F20										
Voltage (keV)	200										
Magnification	50,000 x										
Total dose (e^-/Å^2)	30										
Micrographs collected	54	54	61	56	57	58	64	55	56	55	55
Pixel size (Å)	1.732										
Initial number of particles	65,897	97,728	111,929	96,971	104,714	103,866	109,150	98,131	98,540	93,279	92,277
Number of particles used for EM map building	39,122	25,688	3,985	35,869	1,894	52,784	48,639	21,039	50,625	44,106	52,181
Symmetry	C1										

References

1. Yurkovetskiy, L., Wang, X., Pascal, K. E., Tomkins-Tinch, C., Nyalile, T. P., Wang, Y., Baum, A., Diehl, W. E., Dauphin, A., Carbone, C., Veinotte, K., Egri, S. B., Schaffner, S. F., Lemieux, J. E., Munro, J. B., Rafique, A., Barve, A., Sabeti, P. C., Kyratsous, C. A., Dudkina, N. V., Shen, K., and Luban, J. (2020) Structural and Functional Analysis of the D614G SARS-CoV-2 Spike Protein Variant. *Cell* **183**, 739-751 e738
2. Yan, R., Zhang, Y., Li, Y., Ye, F., Guo, Y., Xia, L., Zhong, X., Chi, X., and Zhou, Q. (2021) Structural basis for the different states of the spike protein of SARS-CoV-2 in complex with ACE2. *Cell Res* **31**, 717-719
3. Zhang, J., Cai, Y., Xiao, T., Lu, J., Peng, H., Sterling, S. M., Walsh, R. M., Jr., Rits-Volloch, S., Zhu, H., Woosley, A. N., Yang, W., Sliz, P., and Chen, B. (2021) Structural impact on SARS-CoV-2 spike protein by D614G substitution. *Science* **372**, 525-530
4. Benton, D. J., Wrobel, A. G., Roustan, C., Borg, A., Xu, P., Martin, S. R., Rosenthal, P. B., Skehel, J. J., and Gamblin, S. J. (2021) The effect of the D614G substitution on the structure of the spike glycoprotein of SARS-CoV-2. *Proc Natl Acad Sci U S A* **118**
5. Gobeil, S. M., Janowska, K., McDowell, S., Mansouri, K., Parks, R., Manne, K., Stalls, V., Kopp, M. F., Henderson, R., Edwards, R. J., Haynes, B. F., and Acharya, P. (2021) D614G Mutation Alters SARS-CoV-2 Spike Conformation and Enhances Protease Cleavage at the S1/S2 Junction. *Cell Rep* **34**, 108630
